# The Spanish Version of the Eating Habits Questionnaire (EHQ-ES) and Its Links to Symptoms and Concerns Characteristic of Eating Disorders among Young Adults

**DOI:** 10.3390/nu13061993

**Published:** 2021-06-10

**Authors:** María Laura Parra-Fernández, María Dolores Onieva-Zafra, Juan José Fernández-Muñoz, Alicja Głębocka, Elia Fernández-Martínez, Anna Brytek-Matera

**Affiliations:** 1 Department of Nursing, Physiotherapy and Occupational Therapy, Faculty of Nursing of Ciudad Real, Universidad de Castilla-La-Mancha, 13001 Ciudad Real, Spain; marialaura.parra@uclm.es; 2 Department of Psychology, University Rey Juan Carlos, 28933 Móstoles, Spain; juanjose.fernmandez@urjc.es; 3 Department of Social Science, Jan Dlugosz University in Czestochowa, 42-200 Częstochowa, Poland; a.glebocka@ujd.edu.pl; 4 Department of Nursing, University of Sevilla, 41009 Sevilla, Spain; efmartinez@us.es; 5 Institute of Psychology, University of Wroclaw, 50-527 Wroclaw, Poland

**Keywords:** orthorexia nervosa, EHQ-ES, validation, EFA, CFA

## Abstract

The aim of the study was to investigate the psychometric properties (internal consistency, factor structure, and convergent validity) of the Spanish version of the Eating Habits Questionnaire (EHQ-ES) and its links to disordered eating in young adults. Two university student samples with a total of 487 participants (*N1*_age_ = 21.75 ± 5.10; *N2*_age_ = 21.17 ± 6.81) participated in the study. They filled out the Eating Habits Questionnaire and the Eating Attitudes Test. Our findings showed that the EHQ-ES demonstrated strong internal consistency, with a Cronbach’s α = 0.903 and ω = 0.939. The EHQ-ES consists of 20 items to measure problems associated with healthy eating (α = 0.855, ω = 0.879), knowledge of healthy eating (α = 0.783, ω = 0.826) and feeling positively about healthy eating (α = 0.775, ω = 0.773). In addition, subscales of the EHQ correlated with subscales of the EAT-26, showing that Orthorexia Nervosa is associated with disordered eating in a sample of university students. The current study is the first to describe a Spanish version of the EHQ. This demonstrates that EHQ-ES is a reliable screening tool for Spanish-speaking young adults. Moreover, the EHQ-ES can be a useful instrument for assessing ON in research and clinical practice.

## 1. Introduction

Recently, there has been an increasing emphasis in society about the importance of food in human health [1]. The involvement of the population in decision making as to the quality and quantity of the food they should and want to include in their diet has become important in everyday life [2]. In this context, a new role appears for the individual as an active agent, manager and producer of their own nutritional health. At the same time and due to the promotion of healthy behaviors in general, society is paying more attention or even “excessive attention” to healthy eating [3,4]. In this new scenario, a potential disorder emerges, orthorexia nervosa (ON), which was first defined as an extreme form of healthy food consumption [5]. Despite the fact that ON is not yet included in the Diagnostic and Statistical Manual of Mental Disorders (DSM-5) and despite the lack of accepted diagnosis criteria, an extreme adherence to this form of healthy eating can lead to individuals experiencing serious physical and psychological consequences that affect their quality of life, including social withdrawal, loss of independence, diminished self-esteem and depression [6,7,8]. Most individuals with ON report discomfort and feel a higher level of anxiety and panic during mealtimes, which affects their interactions with family and non-family members [8,9].

Typically, self-reported scales are used to measure ON or the risk of ON. Such tools are easy to implement, need little time to complete and provide an individual’s perspective on how their eating problems affect their daily lives [10]. They can provide valuable information to health services to help them direct management decisions on this problem [11]. In addition, nowadays, there is a growing realization that the focus on prevention and health promotion of individuals and the results reported in studies using these methods are of great importance in health care research and subsequent treatment. Previous studies [12,13,14] have found that ON is related to “eating disorder risk” in young adults; thus, many of them also assessed the relationship between ON and symptoms and concerns characteristic of eating disorders.

To measure ON, the scientific community has developed different measuring instruments [3,10,15,16]. The assessment of ON is closely dependent on the quality of the measurement methods. According to recent research, there are different instruments available to assess different aspects of ON, but not all of them have been formally evaluated for their reliability, validity and responsiveness [17]. The ORTO-15 has been one of the most used and translated tools to assess ON worldwide, and despite the large number of studies and translations of this scale into several languages [11,18,19,20,21], the scientific community has concluded that this tool lacks an acceptable model and showed low internal reliability and low correlation with the other instrument tools [22,23,24,25]. Therefore, in consideration of other available questionnaires, the use of the Eating Habits Questionnaire (EHQ) and the Düsseldorf Orthorexia Scale (DOS), which seem to be reliable assessments of ON symptomatology, has been recommended [7,17,26].

The EHQ [27] is a questionnaire that was initially created with 160 items to finally become a self-report instrument with 21 items, divided into three factors: (1) Knowledge of healthy eating with 5 items, (2) Problems associated with healthy eating with 12 items and (3) Feeling positively about healthy eating with 4 items. Items are scored on a four-point scale ranging from 1 (false, not at all true), 2 (slightly true), 3 (mainly true) to 4 (very true). The scores for individual subscales and the total scale were summed, with higher scores indicating increased ON tendencies. The authors of the EHQ demonstrated the validity of the questionnaire, finding its scores positively correlated with higher levels of disordered eating behaviors. Previous studies that have been conducted with different measures of ON symptomatology found these same correlations in order to clarify if ON has more in common with eating disorders, if it is a new disorder or a subtype of eating disorder [8,28].

It is worth pointing out the EHQ has never been translated and validated in a Spanish population. We believe it is innovative to validate the latest instrument used in ON studies, which seems to be closer, according to the scientific community, to an assessment of ON symptomatology, and which will therefore be very useful in the advances in the detection of this pathology in the Spanish-speaking community. Therefore, the objective of this study was to evaluate the psychometric properties (internal consistency, factor structure and convergent validity) of the Spanish version of the EHQ among young adults. The previous studies [24,27,28] found that ON is related to “eating disorder risk” in young adults; thus, we also assessed the relationship between symptoms associated with ON and symptoms commonly linked to eating disorders in our sample study.

## 2. Materials and Methods

### 2.1. Study Participants

This study was a cross-sectional survey with a total sample of 487 university students from the University of Castilla–La Mancha, Spain. Participants (*N* = 487) were recruited online during classes from April 2019 to January 2020. The participants completed the questionnaires voluntarily using the electronic platform “Google form” and according to the principles of the Helsinki Declaration. Data handling issues encompassed electronic systems (computer workstations and laptops) for storage, archival, sharing, and disposing off data. All the participants responded voluntarily to the survey and did not receive any financial compensation for participation in the study. Their anonymity was guaranteed and they completed their informed consent before starting the survey.

The study was approved by the Ethics Committee of the University Hospital of Castilla–La Mancha, in accordance with the ethical guidelines established by the Declaration of Helsinki in 2008 (Code C-270).

### 2.2. Measures

#### 2.2.1. Assessment of the Symptoms and Concerns Characteristic of Eating Disorders: The EAT-26

The Eating Attitudes Test-26 [29], originating from the EAT-40 [30], is a 26-item tool assessing “eating disorder risk”. In our study, we used the Spanish version of the EAT-26 [31,32]. Each question had 6 response options (never, rarely, sometimes, often, very often, always); the first 3 were scored with 0, the fourth with 1, the fifth with 2 and the sixth with 3. The total score is the sum of the values of the items, taking into account that question 25 is scored inversely: the higher the score, the higher the risk of eating disorder. In the Spanish version of the EAT-26, there are 4 underlying domains: bulimia, dieting, preoccupation with food and oral control. Cronbach’s alpha was 0.90 for the Spanish version [32]. In the present study, Cronbach’s alpha also was 0.90.

#### 2.2.2. Assessment of Orthorexia Nervosa: The EHQ

The EHQ [26] is a multidimensional measure of ON symptomatology with factors measuring: (1) knowledge of healthy eating, (2) problems associated with healthy eating, and (3) feeling positively about healthy eating. Items are on a 4-point scale (false, not at all true, slightly true, mainly true, very true), where higher scores are indicative of ON tendencies. Adequate internal consistency across all three subscales (α = 0.82 to 0.90) and 2- to 4-week test–retest reliability (r = 0.72 to 0.81) were reported [26].The Cronbach’s alpha in the current sample was α = 0.94 for the total scale, and α = 0.82, 0.89, and 0.73, respectively, for the knowledge, problems, and feelings subscales.

The cross-cultural adaptation includes cultural and linguistic adaptations of the questionnaire in addition to examining the psychometric properties of reliability and validity [31]. Prior to beginning the process, we received approval from the first author of the original scale (application approved in May 2019 by Gleaves). The process was begun in accordance with the international standards published by Beaton et al. [33]. The EHQ English questionnaire was translated back and forth from English to Spanish independently by subject matter and professional linguistic experts. To ensure content validity, a harmonization meeting was held to combine the two versions of the translated questionnaire, composed of a multidisciplinary team of experts. The adaptation process took into account barrier factors in linguistic comprehension, contextualized meaning associated with a construct and possible interpretations of the translated instrument. The final version of the Spanish EHQ-ES (Table 1) was then tested with four members of the target population (university students). This process ensured content and questionnaire validity and the comprehensibility of all items. No further modifications were necessary.

### 2.3. Statistical Analysis

The whole sample (*N* = 487) was randomly split into two independent subsamples. We applied an Exploratory Factor Analysis (EFA) on the first subsample (*N*_1_ = 286) and a Confirmatory Factor Analysis (CFA) on the second subsample (*N*_2_ = 201). According to different authors [34,35], it is most appropriate, if the sample is considered as a large sample, to randomly split the sample in half. An EFA to identify the number of factors is conducted on the first half. Once the EFA reveals the best number of factors to represent the data, then a CFA is conducted on the second random half to confirm that this new factor structure fits the data [34,35].

With the first sample, an Exploratory Factor Analysis (EFA) was applied using SPSS 25 (IBM-SPSS, Armonk, NY, USA, 2017). Before the statistical analysis, data assumptions such as high sample size, multivariate normality, linearity and correlation between variables were tested; Kaiser–Meyer–Olkin (KMO) index and the Barlett test of Sphericity were the criteria (REF); to identify the adequate factor numbers, Cattell’s scree plot and Horn’s parallel analysis were applied with the first sample [36].

A Confirmatory Factor Analysis (CFA) was applied using AMOS 19.0 with a second sample (*N*_2_ = 201). We tried to confirm the exploratory solution and for this, the overall fit was tested using the following fit indices: the Chi-Square statistic X2, the Adjusted goodness of fit index (AGFI), Comparative Fit Index (CFI), Normed Fit Index (NFI), Tuckey–Lewis Fit Index (TLI). In these indices, the reference value is >0.90 to consider an acceptable model (range between 0 and 1); for parsimony adjustment, Root Mean Square Residual (RMSR) and Root Mean Square Error of Approximation (RMSEA) whose reference values are <0.06 [37] and Akaike Information Criterion [38,39].

## 3. Results

### 3.1. Characteristics of the Samples

#### 3.1.1. Sample 1

Concerning the EFA, the analyses were focused on a sample of 286 nursing students from a public Spanish University. In the first sample, 81.9% were female and 18.9% were male. The mean age was 21.75 years (SD = 5.10). The mean Body Mass Index (BMI) was 22.61 kg/m^2^ (SD = 3.61, with mean weight of 63.78 kg (SD = 13.03) and mean height of 167.83 cm (SD = 8.14). Moreover, 56.9% of the nursing students had gone on a diet during the last year and 43.1% had never done a diet.

#### 3.1.2. Sample 2

The second sample was composed of 201 nursing students. In the second sample, 52.2% were female and 47.8% were male. The mean age was 21.17 years (SD = 6.81). The mean BMI was 23.83 kg/m^2^ (SD = 2.85, with mean weight of 70.54 kg (SD = 12.26) and mean height of 171.65 cm (SD = 0.099). In addition, 65.2% had gone on a diet during the last year and 34.3% had not.

### 3.2. Exploratory and Confirmatory Factor Analysis

Table 2 summarizes the factorial solution of the EHQ, factorial weights, percentage of variance explained, Cronbach’s alpha and coefficient Omega. The number of factors was three, similar to the original instrument by Gleaves, Graham and Ambwani [27]. The first factor, named problems, was composed of nine items, for instance, “I only eat what my diet allows” or “I go out less since I began eating healthily”. The second factor, called knowledge, was composed of eight items, such as, “I follow a diet with many rules” or “I turn down social offers that involve eating unhealthy food”. The third factor, named feeling, consisted of three items, for instance “I feel in control when I eat healthily” or “I prepare food in the most healthful way”. The iterative process of item removal to improve model fit led to the suppression of one item: EHQ19, i.e., “I follow a health-food diet rigidly”. This item in the original test belongs to the feeling “dimension” but it has a factor weight of 0.614 on the problems dimension and 0.449 on the knowledge dimension and has no factor weight in its original feeling dimension. Furthermore, the skewness value was 1.73, the kurtosis value was 2.38 and the mean was 1.40 in a range of 1–4, which justifies its elimination.

Regarding reliability, the overall Cronbach’s alpha was 0.903 and Omega was 0.939. For each factor, the findings were: α = 0.855, ω = 0.879 for the first factor; α = 0.783, ω = 0.826 for the second factor; and α = 0.775, ω = 0.773 for the third factor. These indices can be interpreted as excellent and coherent with the internal consistency of the original questionnaire. Moreover, the homogeneity indices range was between 0.363 (item 2) and 0.636 (item 21).

According to the exploratory solution, a CFA was conducted on a second sample in order to confirm the fit of the model. The goodness-of-fit indices’ global scale was: X^2^ = 279.26, *p* = 0.000 (df = 156), Adjusted Goodness of Fit index = 0.831, Comparative Fit Index = 0.911, Normed Fit Index = 0.835, Tucker–Lewis Fit Index = 900, Root Mean Square Residual = 0.051, Root Mean Square Error of Approximation = 0.065 and Akaike Information Criterion was 387.268.

### 3.3. Convergent Validity

As presented in Table 3, the convergent validity of the EHQ was significantly correlated with EAT-26 and its dimensions. The correlations between three subscales of the EHQ (problems, knowledge and feeling) and four subscales of the EAT-26 (bulimia, diet, worry about food and control oral) were significant with a confidence level of *p* < 0.01. Only the correlations between oral control and knowledge (r = 0.084, *p* > 0.05) and feelings (r = 0.050, *p* > 0.05) were not significant.

## 4. Discussion

This study aimed to evaluate the psychometric properties of the Spanish version of the EHQ (EHQ-ES) in young adults. The EHQ-ES demonstrated strong internal consistency with a Cronbach’s α = 0.903 and ω = 0.939. In other EHQ versions [12,40,41], the Cronbach’s α ranged from 0.75 to 0.90. The EHQ-ES consists of 20 items and has a similar factorial structure to the original version of the EHQ [27] as well as the Italian version [42]. The Polish version [12], compared to the original scale, had the same number of factors but a reduced number of items (14 items instead of 21). On the other hand, the Hungarian version of the EHQ [37] revealed four factors that the authors named differently (thoughts about healthy eating, dietary restriction, diet superiority and social impairment) but none of the items were eliminated. The French version of the EHQ [36] was reduced to 16 items in a three-factor structure. Although the psychometric properties of the different versions analyzed show promising results, the discussion among the scientific community is regarding the quality of the measurement model of ON [17,41]. In the English version of the EHQ [43], the authors named one of the dimensions “Behaviours-subscale” as a result of the loading of three items, belonging in the original scale to the dimension of “problems”, into the dimension “knowledge”; as a consequence, there is a change in the definition of this dimension [41].

Likewise, there are differences in the variability of the number of items eliminated among the different versions, and, in certain cases, there are similarities among the different versions. In relation to other validated ON tests, in which there is also this difference or shift of items from one dimension to another, some authors suggest that it may be due to sociocultural differences, taking into account the social aspect surrounding meals [8,11]. However, there are authors who accept this movement of items in terms of test structure or reliability [25]. In our study, within the information related to the EFA results, the item EHQ19 (“I follow a health-food diet rigidly”) was removed because it had no factorial weight on any dimension. According to different authors, a translated instrument needs to be revalidated with the target population because of the potential distortions of the items during the translation procedure [44,45]. However, according to other authors, instrument validation in cross-cultural research is evaluated with the assessment of multiple psychometric properties; scale means, variance, reliabilities, criterion validity and factorial validity are appropriate approaches for instrument validation [46]. The cultural differences and the translation procedure often induce the failure to obtain invariance of the psychometric properties of the original and the translated version of the instrument in cross-cultural research. The reliability and validity of the measures are main indicators of the quality of a measuring instrument and while the complexity of translation should not be underestimated, not having well-defined diagnostic criteria may alter the value of the meaning or weight of the measure in terms of what the questionnaire is supposed to measure.

According to the third factor of the EHQ (feelings), the factorial solution of the CFA showed that item 15 and item 21 did not have a significant weight on the construct feeling. Moreover, in the EFA, the factorial weights were almost similar to second factor (knowledge), in this sense, the items meaning could be related by the participants with the knowledge dimension. In fact, both items show actions related with eating habits; specifically, it involves actions that people make in order to eat healthy. Therefore, it would not be related directly with their feelings.

Regarding divergent validity, the correlational analysis of the EHQ-ES and the EAT-26 revealed that coefficient correlations ranged from 0.163 to 0.645. Our results showed that a higher score on the EHQ-ES designed to detect ON symptoms correlated with a higher score on the EAT-26, elevated symptoms and concerns characteristic of eating disorders in a sample of university students. Gleaves et al. [27] also found a relationship between problems (r = 0.79), knowledge (r = 0.54) and feelings (r = 0.41) and pathological eating (EAT-26 total score). In addition, problems associated with healthy eating in the original EHQ correlated more highly with measures of eating pathology (EAT-26) than with measures of general pathology, personality, or social desirability in a college student sample [27]. Our findings were consistent with previous studies [12,40,41,42] that found no significant relationship between feeling positively about healthy eating and self-control about food and social pressure to gain weight (oral control). It is worth adding that the Italian version of the EHQ total score correlated with eating pathology as well (measured by the Eating Disorder Inventory-3-Referral Form) [42]. To sum up, our results are consistent with numerous studies [27,43,47,48] showing that greater ON symptomatology is associated with higher levels of disordered eating.

Our study has some limitations. The first is that our sample was a convenience sample and was comprised of young adults (university students). This highlights the lack of generalizability of the current findings. Another limitation is the fact that as they are all nursing students and, therefore, more focused on health, this knowledge may indirectly affect their responses as they were more likely to have healthy eating habits. Nevertheless, our samples have very similar demographic characteristics to the original sample where the questionnaire was created. Therefore, it is important that psychometric evaluation of the EHQ-ES is repeated in a general population Moreover, a large sample size would provide more conclusive evidence regarding the psychometric properties of the EHQ-ES. Another limitation of the present study is selection bias (due to the non-representative nature of the respondents, also called the ‘volunteer effect’) [49]. Finally, due to the cross-sectional study design, test–retest reliability was not assessed in the present study. Notwithstanding these limitations, the study suggests that the EHQ-ES displays significant validity.

## 5. Conclusions

The EHQ-ES appears to be a valid and reliable instrument to assess cognition, behaviors, and feelings related to ON in Spanish-speaking young adults. The EHQ-ES can be a useful instrument for assessing ON in research and clinical practice.

## Figures and Tables

**Table 1:** The Spanish version of the EHQ (EHQ-ES).

1. Estoy más informado/a que los demás acerca de la alimentación saludable. 1. I am more informed than others about healthy eating.
2. Rechazo ofertas sociales que conllevan tener que comer comida poco sana. 2. I turn down social offers that involve eating unhealthy food.
3. La forma en la que preparo mi comida es importante en mi dieta. 3. The way my food is prepared is important in my diet.
4. Sigo una dieta que tiene muchas reglas. 4. I follow a diet with many rules.
5. Mis hábitos alimenticios son superiores a los de los demás. 5. My eating habits are superior to others.
6. Me distraigo con pensamientos de comer de forma saludable. 6. I am distracted by thoughts of eating healthily.
7. Solo como lo que mi dieta me permite. 7. I only eat what my diet allows.
8. Comer de forma saludable es una fuente significativa de estrés en mis relaciones. 8. My healthy eating is a significant source of stress in my relationships.
9. He hecho muchos esfuerzos para comer de forma más saludable a lo largo del tiempo. 9. I have made efforts to eat more healthily over time.
10. Mi dieta afecta el tipo de empleo que yo aceptaría. 10. My diet affects the type of employment I would take.
11. Mi dieta es mejor que las dietas de los demás. 11. My diet is better than other people’s diets.
12. Me siento en control cuando como de forma saludable. 12. I feel in control when I eat healthily.
13. El año pasado, los amigos o miembros de la familia me han comentado que estoy demasiado/a preocupado/a con comer sano. 13. In the past year, friends or family members have told me that I am overly concerned with eating healthily.
14. Tengo dificultad para encontrar restaurantes que sirvan las comidas que yo como. 14. I have difficulty finding restaurants that serve the foods I eat.
15. Comer de la manera que yo lo hago me da una sensación de satisfacción. 15. Eating the way I do gives me a sense of satisfaction.
16. Pocas comidas son saludables para que yo las coma. 16. Few foods are healthy for me to eat.
17. Me paso más de tres horas al día pensando en comer de forma saludable. 17. I go out less since I began eating healthily.
18. Salgo menos desde que he empezado a comer de forma saludable. 18. I spend more than three hours a day thinking about healthy food.
19. Sigo de forma rígida una dieta de comida saludable. 19. I follow a health-food diet rigidly
20. Me siento genial cuando como de forma saludable. 20. I feel great when I eat healthily.
21. Preparo comida de la forma más saludable. 21. I prepare food in the most healthful way.

**Table 2:** Factorial solution of Eating Habits Questionnaire (EHQ-21).

Items	EFA First Sample			CFA Second Sample		
	P	K	F	P	K	F
EHQ18: I spend more than three hours a day thinking about healthy food	0.706			0.585		
EHQ13: In the past year, friends or family members have told me that I am overly concerned with eating habits	0.666			0.714		
EHQ8: My healthy eating is a significant source of stress in my relationship	0.660			0.549		
EHQ17: I go out less since I began eating healthily	0.652			0.586		
EHQ7: I only eat what my diet allows	0.626	0.322		0.544		
EHQ14: I have difficulty finding restaurants that serve the food I eat	0.608			0.762		
EHQ10: My diet affects the type of employment I would take	0.569			0.578		
EHQ16: Few foods are healthy for me	0.540			0.654		
EHQ6: I am distracted by thoughts of eating habits	0.457		0.435	0.523		
EHQ5: My eating habits are superior to others		0.768			0.577	
EHQ11: My diet is better than other people’s diet		0.768			0.500	
EHQ3: The way my food is prepared is important in my diet		0.518	0.480		0.620	
EHQ1: I am more informed than others about healthy eating		0.514			0.553	
EHQ4: I follow a diet with many rules	0.482	0.513			0.544	
EHQ2: I turn down social offers that involve eating unhealthy food		0.425			0.395	
EHQ20: I feel great when I eat healthily.			0.755			0.611
EHQ12: I feel in control when I eat healthy			0.666			0.577
EHQ9: I have made efforts to eat more healthily over time			0.653			0.627
EHQ21: I prepare food in the most healthy way		0.520	0.634			
EHQ15: Eating the way I do gives me a sense of satisfaction		0.485	0.530			
Variance explained	35.53	8.63	6.45			
Cronbach’s Alpha	0.855	0.783	0.775			
Omega	0.879	0.826	0.773			

Note: Factorial weight less than 0.30 were excluded; P = Problems; K = Knowledge; F = Feelings.

**Table 3:** Correlations of the EHQ and its dimension with the EAT-26 dimensions.

	EHQ Total	EHQ Problems	EHQ Knowledge	EHQ Feelings
Bulimia	0.565 **	0.645 **	0.379 **	0.398 **
Dieting	0.644 **	0.595 **	0.515 **	0.537 **
Worry about food	0.442 **	0.488 **	0.242 **	0.411 **
Oral control	0.163 **	0.257 **	0.084	0.050
EAT-26 total	0.582 **	0.629 **	0.391 **	0.459 **

** *p* < 0.01.

## Data Availability

The dataset used during the current study is available from the corresponding author on reasonable request.
